# Possibility of Japanese Cedar Pollen Causing False Positives in the Deep Mycosis Test

**DOI:** 10.3390/ijms22042135

**Published:** 2021-02-21

**Authors:** Takashi Kanno, Changmin Kim, Daisuke Yamanaka, Ken-ichi Ishibashi, Hiroshi Tanaka, Naohito Ohno, Yoshiyuki Adachi

**Affiliations:** 1 Laboratory for Immunopharmacology of Microbial Products, School of Pharmacy, Tokyo University of Pharmacy and Life Sciences, 1432-1 Horinouchi Hachioji, Tokyo 192-0392, Japan; kannotak@toyaku.ac.jp (T.K.); y151059@toyaku.ac.jp (C.K.); ymnkd@toyaku.ac.jp (D.Y.); ohnonao@toyaku.ac.jp (N.O.); 2 Department of Host Defense and Responses, Kagawa Nutrition University, 3-9-21 Chiyoda, Sakado, Saitama 350-0288, Japan; ishibashi.kenichi@eiyo.ac.jp; 3 Department of Chemical Science and Engineering, Tokyo Institute of Technology, 2-12-1-H101, Oookayama, Meguro, Tokyo 152-8552, Japan; thiroshi@cap.mac.titech.ac.jp

**Keywords:** beta-d-glucan, glucan-binding protein, Limulus amebocyte lysate assay, pollen

## Abstract

Because Japanese cedar pollen (JCP) contains beta-1,3-d-glucan (BG), there is concern that its lingering presence in the atmosphere, especially during its scattering period, may cause false positives in the factor-G-based Limulus amebocyte lysate (LAL) assay used to test for deep mycosis (i.e., G-test). Hence, we examined whether the LAL assay would react positively with substances contained in JCP by using the G-test to measure JCP particles and extracts. BG was purified from the JCP extract on a BG-specific affinity column, and the percentage extractability was measured using three different BG-specific quantitative methods. The G-test detected 0.4 pg BG in a single JCP particle and 10 fg from a single particle in the extract. The percentage extractability of JCP-derived BG was not significantly different among the three quantitative methods. As the JCP particles should technically have been removed during serum separation, they should be less likely to be a direct false-positive factor. However, given that the LAL-assay-positive substances in the JCP extract were not distinguishable by the three BG-specific quantitative methods, we conclude that they may cause the background to rise. Therefore, in Japan false positives arising from JCP contamination should be considered when testing patients for deep mycosis.

## 1. Introduction

Beta-1,3-d-glucan (BG) is an important polysaccharide that occurs naturally in the cell walls of most fungi as well as some bacteria and higher and lower plants. In mammals, fungal BG is recognized as a pathogen-associated molecular pattern by the host’s immune system, where it binds to the host’s C-type lectin receptor Dectin-1 and induces immune processes such as inflammation and phagocytosis [1,2,3,4]. The immune response mediated through Dectin-1 depends on the type and properties of the BG. For example, the algal BG laminarin (LAM) was found to be a Dectin-1 antagonist, suppressing immunological reaction by fungal BG and fungal-BG-related colitis in the intestine of mice [5]. Similar BG-binding proteins exist in other species, such as insects. For example, in the blood lymph of silkworms, a complement-like protein called BG recognition protein (BGRP) induces immune responses such as melanization and the production of antibacterial peptides in response to fungal BG [6].

Fungal infections can be classified according to the site of infection; that is, superficial, cutaneous, subcutaneous, or systemic (deep) mycosis. Currently, the auxiliary diagnosis of deep mycosis is detected with the Limulus amebocyte lysate (LAL) assay using coagulant factor G [7,8,9,10]. Factor G is a serine protease zymogen that is present on the surface of blood cells of the horseshoe crab, where it responds to fungal BG by activating a coagulation reaction system to eliminate the contaminant [11]. Although the LAL assay is highly sensitive, several factors can cause it to generate false-positive results. For example, BGs with a β-1,4-d-glucan chain, which are present in medical gauze and cellulose dialysis membranes, are risk factors for generating false positives in the assay [12,13,14]. Schizophyllan (SPG) and lentinan (LNT), which are fungal BGs used as main ingredients in some medicinal preparations, will naturally give LAL-assay-positive results [15]. Some antibacterial, antiviral, antifungal, or blood products have also been reported to contain LAL-assay-positive substances [16,17,18]. These medicines and medical devices are often used in patients who are at high risk of developing deep mycosis. Moreover, various sources of BG (e.g., airborne and resident skin fungi and bacteria, pollen, and dust) present in the sampling environment can also contribute to false positives [19].

Pollen contains BG as it uses this polysaccharide for germination [20,21,22]. Therefore, pollen particles and extracts from several plants have been found to contain LAL-assay-positive substances and allergens; however, these positive results are speculated to be caused by the pre-existing BG in pollen [23,24]. Although various types of pollen are scattered around Japan throughout the year, the most widely distributed is Japanese cedar pollen (JCP), which can be deposited at a rate of 1.2 × 10^9^ particles/m^2^ during a 2 month period [25]. Recently, we reported that 1 g of JCP contains 2 μg of LAM-equivalent BG, which can contribute to the stimulation of allergen-specific immunity via Dectin-1 [26]. Generally, the average weight of JCP is 12 ng [27]; therefore, the BG content of one JCP particle was estimated from this present study to be 24 fg. Therefore, if 1 mL of any test sample is contaminated with approximately 800 particles of JCP, the BG content may exceed the cutoff value of the LAL assay. Additionally, if the LAL assay reacts with other substances in JCP, such as cellulose, then even a smaller amount of JCP contamination could cause false-positive results.

Therefore, the objective of this study was to clarify whether the various components of JCP, particularly its BG, could be false-positive substances for the LAL assay. Several BG probes were tested; namely, the soluble protein mouse Dectin-1 (mDectin-1), an artificial BGRP (supBGRP), and Limulus factor G. Our findings have clinical relevance in ensuring that patients in Japan receive an accurate diagnosis of deep mycosis from the LAL assay, especially during the high JCP scattering period.

## 2. Results

### 2.1. Comparison of the Specificities of supBGRP and mDectin-1

**Figure 1:**
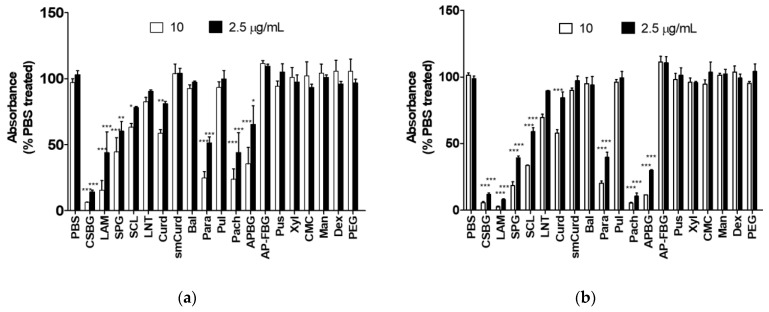
Specificity of two different beta-1,3-d-glucan (BG) probes to various polysaccharides. The specificity was determined from the inhibitory activity of the tested polysaccharides on probe binding to scleroglucan. (**a**) Specificity of supBGRP. (**b**) Specificity of mDectin-1. Results are shown as the mean ± SD (*n* = 3). The absorbance of each sample treated with the probe (2.5 or 10 μg/mL) was compared with that of the PBS-treated sample using Dunnett’s multiple comparison test; * *p* < 0.05, ** *p* < 0.01, *** *p* < 0.001. APBG, *Aureobasidium pullulans* beta-glucan; AP-FBG, fermented beta-glucan from *A. pullulans*; Bal, barley beta-glucan; CSBG, *Candida* solubilized beta-glucan; CMC, carboxymethylcellulose; Curd, curdlan; Dex, dextran; LAM, laminarin; LNT, lentinan; Man, mannan from *Saccharomyces cerevisiae*; mDectin-1, mouse Dectin-1; Pach, pachyman; Para, paramylon; PBS, phosphate-buffered saline; PEG, polyethylene glycol; Pul, pullulan; Pus, pustulan; SCL, scleroglucan; SPG, schizophyllan; supBGRP, artificial beta-glucan recognition protein; Xyl, xylan from corn core.

The probe supBGRP, which binds to triple-helical BG, is based on the carbohydrate-binding domain sequence of BGRPs from several insects [28]. As shown in Figure 1, supBGRP (Figure 1a) had similar reactivity to mouse Dectin-1 with the various polysaccharides tested (Figure 1b). Both probes were reactive to BGs with a β-1,6-side chain, such as *Candida* solubilized BG (CSBG) [29], LAM [30], pachyman (Pach) [31], SPG [32], *Aureobasidium pullulans* BG (APBG) [33,34], and scleroglucan (SCL) [32].

However, the probes were not reactive to BGs with a high ratio of β-1,6-side chains, such as the fermented BG from *Aureobasidium pullulans* (AP-FBG) [35]. Although both probes were less reactive to the linear BGs, such as curdlan (Curd) [36] and the digested curdlan (smCurd), they were slightly more reactive to those with a large molecular weight, such as paramylon (Para) [37]. Neither of the probes were reactive to BGs with a β-1,4-d-glucan chain, such as that from barley (Bal) [38]; to β-1,4-d-glucan, such as carboxymethylcellulose (CMC); to β-1,6-d-glucan, such as pustulan (Pus) [39]; to α-1,4- and α-1,6-glucan, such as pullulan (Pul) [40]; and to α-1,6-glucan, such as dextran (Dex); as well as to other polysaccharides with different constituent sugars, such as xylan from corn core (Xyl), mannan from *Saccharomyces cerevisiae* (Man), and polyethylene glycol (PEG).

**Figure 2:**
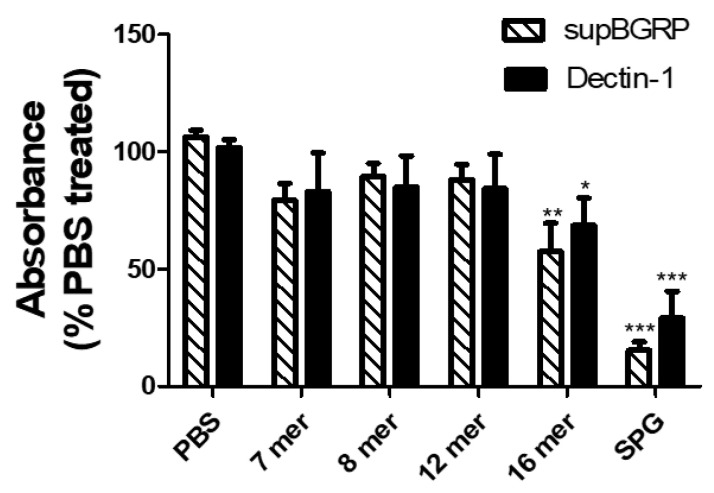
Specificity of beta-1,3-d-glucan probes to various laminari-oligosaccharides. The specificity was determined from the inhibitory activity of the tested oligosaccharides on probes binding to SPG. Results are shown as the mean ± SD (*n* = 3). The absorbance of each sample was compared with that of the PBS-treated sample using Dunnett’s multiple comparison test; * *p* < 0.05, ** *p* < 0.01, *** *p* < 0.001.

To clarify the minimum degree of glucose polymerization that could be bound by the two probes, we examined their reactivity toward various laminari-oligosaccharides of different repeat units (β-1,3-d-glucose polymers). Both supBGRP and mDectin-1 were reactive with the 16-mer oligosaccharide and SPG, which has a β-1,3- main chain of approximately 600 mer. However, both probes did not bind to the 7-, 8-, and 12-mer oligosaccharides (Figure 2).

### 2.2. Effect of pH on the Binding Stability of supBGRP

**Figure 3:**
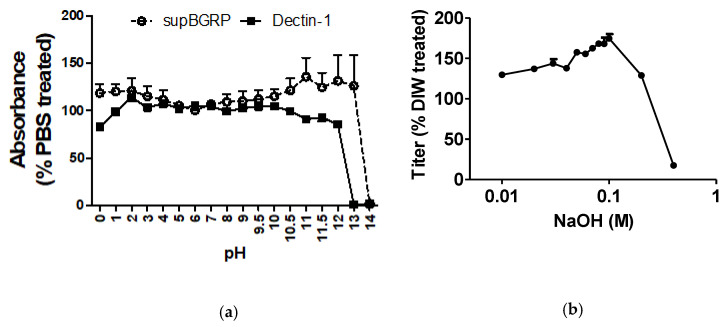

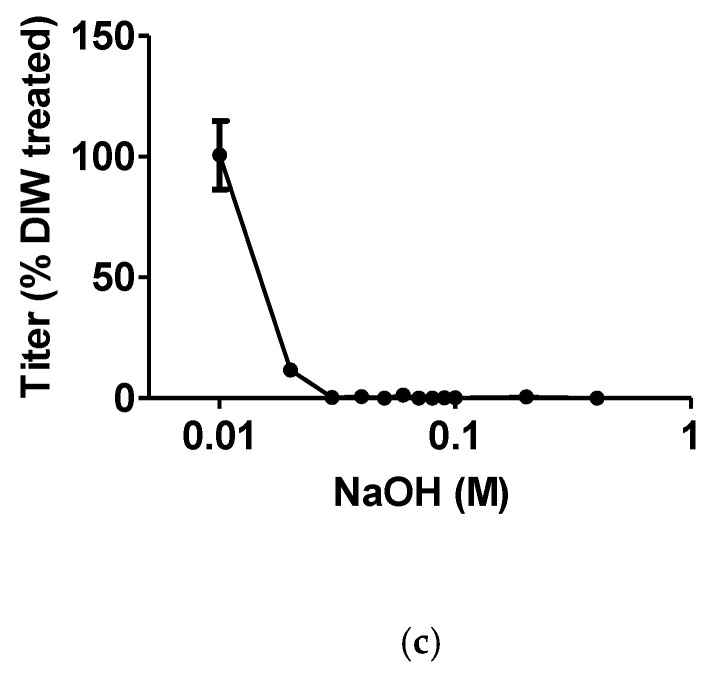
Binding stability of beta-1,3-d-glucan probes. (**a**) Stability of the solid-phase probes (supBGRP and mDectin-1) in several aqueous solutions of varying pH values. (**b**) Stability of solid-phase supBGRP under NaOH treatment. (**c**) Stability of LAM-supBGRP binding under NaOH treatment. Results are shown as the mean ± SD (*n* = 3).

For the comparison of the LAL assay with other diagnostic methods, we used a supBGRP-conjugated affinity column (BGRP column) for concentrating the BGs in the JCP extract or fungal culture. First, we examined the effect of pH on the binding between supBGRP and SCL to clarify the elution conditions of the BGRP column. When the solid-phase supBGRP was treated with a high-pH reagent, the binding of supBGRP to SCL decreased significantly. By contrast, with acid treatment, supBGRP and SCL binding remained at levels similar to those under phosphate-buffered saline (PBS) treatment (Figure 3a). Hence, we further examined the binding dissociation pattern under increasing concentrations of NaOH. The BG captured by the solid-phase supBGRP was released by 0.03 M NaOH treatment (Figure 3b). By contrast, the binding ability of the immobilized supBGRP was not affected, even under treatment with 0.1 M NaOH (Figure 3c).

### 2.3. Column Concentration of Beta-1,3-d-Glucans

**Figure 4:**
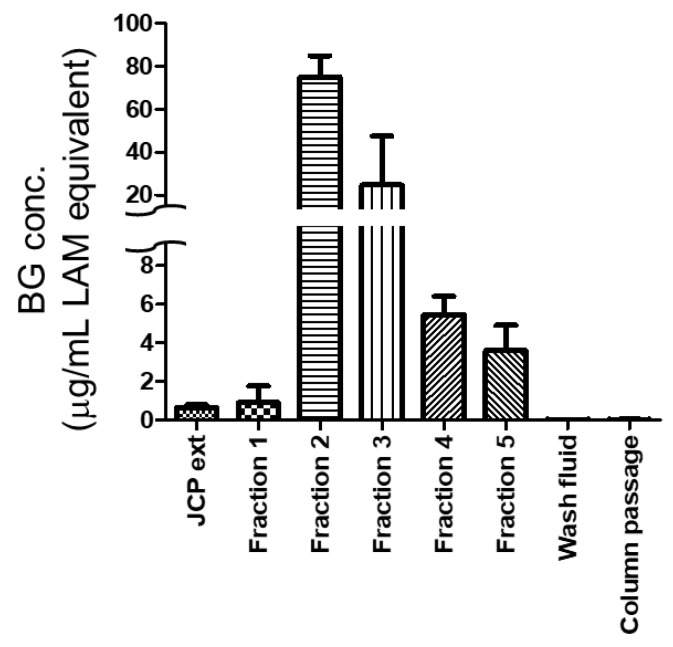
Beta-1,3-d-glucan concentrations in a Japanese cedar pollen extract (JCP ext), each column fraction of the JCP ext, column wash fluids before elution (Wash fluid), and the column passed JCP ext (Column passage). Each sample was quantified by supBGRP enzyme immunoassay, with the data given in terms of the weight concentration of LAM. Results are shown as the mean ± SD (*n* = 3).

**Figure 5:**
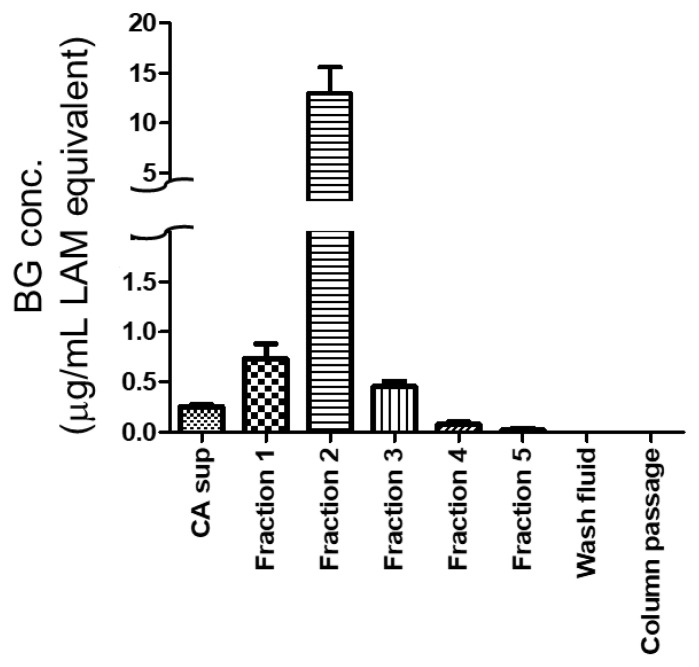
Beta-1,3-d-glucan concentrations in the original *Candida albicans* culture supernatant (CA sup), each column fraction of the CA sup, column wash fluids before elution (Wash fluid), and the column passed CA sup (Column passage). Each sample was quantified by supBGRP enzyme immunoassay, with the data given in terms of the weight concentration of LAM. Results are shown as the mean ± SD (*n* = 3).

The BGRP column was used to concentrate the JCP extract and *Candida albicans* culture supernatant (CA sup). The BG captured by the column was eluted using 900 μL of 0.03 M NaOH per fraction. Almost all the BG molecules were eluted in the second fractions of the JCP extract and CA sup. Moreover, no BG was detected in the wash fluids before and after elution of the BGs through the column (Figure 4 and Figure 5). The non-purified JCP extract contained 600 ng/mL LAM-equivalent BG, whereas fraction 2 eluted from 900 mL of JCP extract contained 75 μg/mL LAM-equivalent BG (Figure 4). By contrast, the CA sup contained 350 ng/mL LAM-equivalent BG, whereas fraction 2 eluted from 100 mL of CA sup contained 13 μg/mL LAM-equivalent BG (Figure 5).

### 2.4. Comparison of Different Beta-Glucan Quantitative Methods

**Figure 6:**
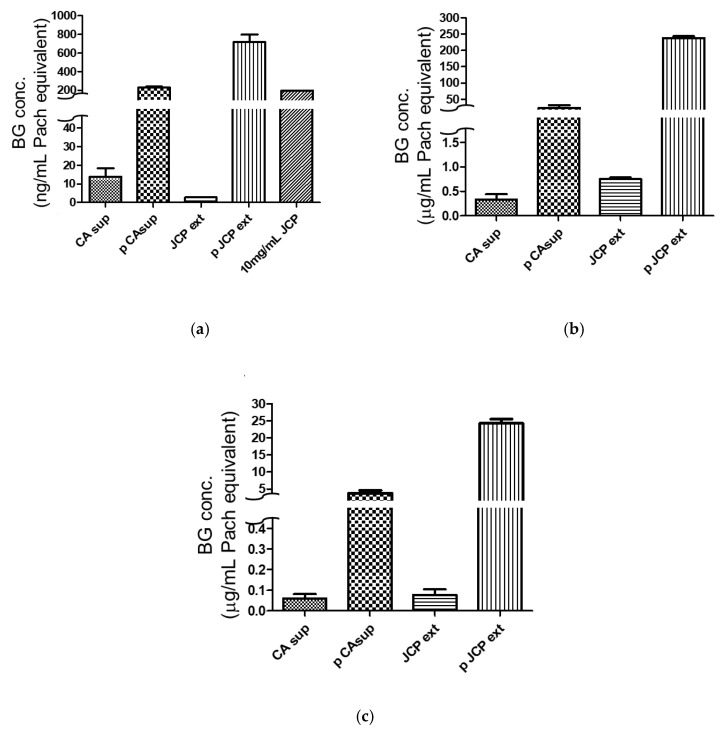
Beta-1,3-d-glucan contents in the BGRP column-purified (p) CA sup and JCP ext, as determined by three quantitative methods. The results are given in terms of pachyman (Pach) equivalents. (**a**) Limulus amebocyte lysate assay using Limulus factor G. (**b**) mDectin-1 enzyme immunoassay (EIA). (**c**) supBGRP EIA. Results are shown as the mean ± SD (*n* = 3).

**Figure 7:**
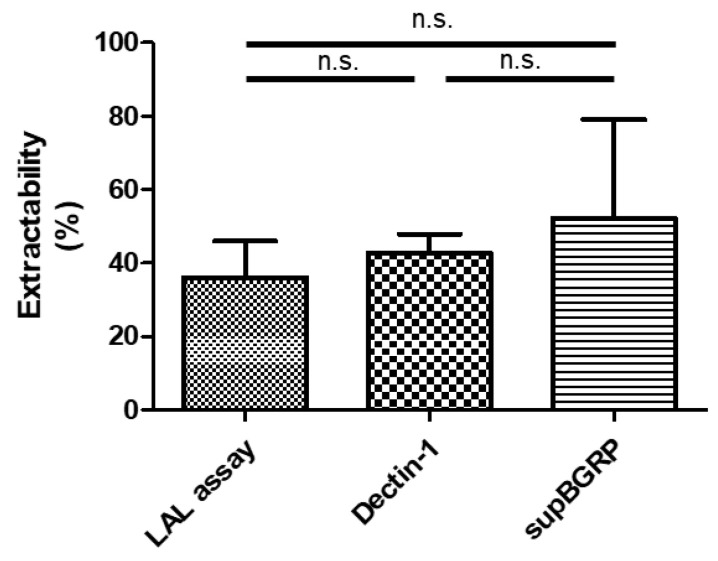
BGRP column extractability of JCP ext. The extractability was determined using the following formula: Extractability (%) = [([BG conc. of purified sample] × [amount of purified sample (= 1.2 mL)])/([BG conc. of JCP ext] × [amount of JCP ext (= 900 mL)]) × 100]. Each sample was quantified by the Limulus amebocyte lysate assay using factor G (LAL assay), the mouse Dectin-1 EIA, and the supBGRP EIA. Results are shown as the mean ± SD (*n* = 3). Data between samples were compared using Tukey’s comparison test; n.s. not significant.

Three BG-specific quantitative methods were tested: an LAL assay using factor G as the probe, an enzyme immunoassay (EIA) using supBGRP as the probe, and an EIA using mDectin-1 as the probe. For all three methods, NaOH-solubilized Pach was used as the standard. As shown in Figure 6a, the LAL assay detected 2.7 ng/mL Pach-equivalent BG in the non-purified JCP extract, 720 ng/mL in the column-purified JCP extract, 13 ng/mL in the CA sup, and 230 ng/mL in the column-purified CA sup (Figure 6a). The Dectin-1 EIA detected 0.74 μg/mL Pach-equivalent BG in the JCP extract, 240 μg/mL in the purified JCP extract, 0.33 μg/mL in the CA sup, and 23 μg/mL in the purified CA sup (Figure 6b). The supBGRP EIA detected 80 ng/mL Pach-equivalent BG in the JCP extract, 23 μg/mL in the purified JCP extract, 60 ng/mL in the CA sup, and 3.7 μg/mL in the purified CA sup. (Figure 6c). Finally, the extractability of the BGRP columns was determined from the pre- or post-column BG amounts (Figure 7). The average extractability percentages were 36.0% for the LAL assay, 42.7% for the mDectin-1 EIA, and 52.1% for the supBGRP EIA. However, no significant differences were noted, with the 95% confidence intervals being −33.94 to 20.58 for LAL assay vs. Dectin-1, −43.33 to 11.19 for LAL assay vs. supBGRP, and −36.65 to 17.87 for Dectin-1 vs. supBGRP.

## 3. Discussion

In this study, we examined whether the substances in JCP could affect the LAL assay to generate false-positive results. The average weight of one JCP particle is 12 ng and 1 mg of JCP contained 8.3 × 10^4^ particles [27]. However, the weight depends on the dryness and growing conditions. The 1 mg of JCP used in this study contained 5 ± 0.2 × 10^4^ particles. After the pretreatment of these particles according to the LAL assay method, the content of test-positive substances was 20 ng/mg JCP. Accordingly, one particle of JCP contained 0.4 pg. The cut-off and normal values for the LAL assay are 20 pg/mL and <10 pg/mL, respectively. Hence, any 1 mL test sample contaminated with 50 JCP particles at the testing stage would constitute a false-positive result in the LAL assay. JCP ruptures rapidly in alkaline or neutral liquids at close to body temperature, releasing soluble-state BGs. Hence, we surmised that serum contaminated with JCP-derived BG at the time of blood sampling would reflect the same condition as the JCP extract. According to the LAL assay, 0.5 ng of test-positive substance was eluted from 1 mg of JCP. Hence, 1 mL of sample contaminated with 2000 JCP particles at the blood collection stage would constitute a false positive in the LAL assay. Previously, it was reported that 15% of the BG released after JCP rupture was water-soluble, whereas the remaining 85% was water-insoluble [26]. Although there were differences in the solubilization and quantitative methods used between the studies, the LAL assay results of this study demonstrated a water-insoluble BG percentage of 96% [=(100 − 100 × [0.5 (ng/1 mg JCP)/12 (ng/1 mg JCP)])(%)], which is higher than the previously reported 85%. This might be due to LAL assay reactive substances other than BG in the water-insoluble fraction. However, in the actual LAL assay, most of the sample preparation steps are performed in a sterile environment. Therefore, one would assume that any JCP particles contaminated in the blood would have been removed at the time of serum separation, and the possibility of false positives attributed to the insoluble BG portion would be quite rare. Hence, the substances in the eluate from the JCP extract that were reactive with the LAL assay were further characterized.

To achieve this, we evaluated the extractability of the JCP extract from the BGRP column using three quantitative methods: the LAL assay and supBGRP and mDectin-1 EIAs. The supBGRP used for the BGRP column is a known stable BG-specific probe. Therefore, if the JCP extract contains a substance that does not react with the BGRP column but reacts with each assay probe (i.e., factor G and mDectin-1), then the calculated extractability would be decreased, indicating that the substance is something other than BG. The reason for using this approach is that the uniform evaluation of BG detective methods is difficult owing to their differences in sensitivity and reactivity to the higher-order structures of BG.

The binding between soluble BG and solid-phase BGRP was found to dissociate at 0.03 M NaOH. Given that the function of supBGRP would not have been affected, the dissociation was deduced to be the result of a structural change in BG. Additionally, the BGRP column could purify BGs from both the CA sup and JCP extract, proving its specificity for this polysaccharide.

As determined with the three quantitative methods, there was no significant difference in the extractability percentages. Hence, it could be concluded that BG was the main substance from the JCP extract that reacted with the LAL assay.

In conclusion, this study clarified the risk of the contribution of JCP to false positives from the LAL assay. Because pollen is negatively charged and adheres easily to glassware, hair, clothes, and skin, it can be inadvertently carried into the indoor environment. According to an academic conference report, 80,000 particles adhered to the clothes of participants walking outside for 1 h during the pollen dispersal period [41]. Given that the false-positive substances in the LAL assay (which are expected to be released into the serum from blood samples contaminated with JCP) could not be distinguished by the different BG detection methods, the prevention of pollen being brought into the room may reduce the risk of false positives from the LAL assay. Our results have particular clinical relevance for deep mycosis testing in Japan, where the atmospheric dispersal of JCP is especially widespread and could affect the accuracy of the test results.

## 4. Materials and Methods

### 4.1. Polysaccharide Reagents

Curd (Wako Pure Chemical Industries, Osaka, Japan), Para (Wako), xylan from corn core (Xyl; TCI, Tokyo, Japan), Pus (Calbiochem, San Diego, CA, USA), Pach (Calbiochem), Bal (Sigma-Aldrich, St. Louis, MO, USA), LAM (Sigma-Aldrich), Man (Sigma-Aldrich), PEG (Sigma-Aldrich), scleroglucan (SCL) (CarboMer, San Diego, CA, USA), Pul (Pfanstiehl, Waukegan, IL, USA), LNT (Yamanouchi, Tokyo, Japan), SPG (Kaken, Tokyo, Japan), CMC (Daiichi Kagaku, Osaka, Japan), and Dextran T10 (Dex; Seikagaku Corp., Tokyo, Japan) were all purchased from the indicated suppliers. APBG was obtained from Kururu (Osaka, Japan) and AP-FBG from ADEKA (Tokyo, Japan). CSBG was obtained as described previously [29]. smCurd was prepared by first digesting Curd with formic acid and then recovering the substances of molecular weight 5 kDa or less by dialysis.

### 4.2. β-Glucan Quantitative Assays

The LAL assay applied was the Fungitec G-test MK 2 “Nissui” (Nissui Pharmaceutical Co., Ltd., Tokyo, Japan), which was performed according to the manufacturer’s protocol. The supBGRP EIA was carried out as previously described [42]. Likewise, mDectin-1 was prepared using a previously described method [43] and used for the mDectin-1 EIA. The standard used for evaluating the column performance was LAM, whereas that used for comparing the quantitative assays was NaOH-solubilized Pach.

### 4.3. Comparing the Specificity and Binding Stability of supBGRP and Dectin-1

In brief, each well of a 96-well half-area microplate (Greiner Bio-One, Kremsmünster, Austria) was coated with 5 μg/mL of SCL in 0.05 M carbonate buffer (pH 9.5) at 4 °C overnight and then blocked with PBS containing 1% bovine serum albumin (Sigma-Aldrich) for 2 h at 25 °C. The coated plate was incubated with 25 μL of 200 ng/mL biotinylated supBGRP or biotinylated mDectin-1 and 25 μL of 10 or 2.5 μg/mL polysaccharide reagents for 2 h at 25 °C on the Taitec Mild Mixer (Taitec, Saitama, Japan). Then, the well components were treated with 1:5000 horseradish-peroxidase-conjugated streptavidin (Biolegend, San Diego, CA, USA) for 1 h at 25 °C. The specificity of the assay was monitored using the TMB Microwell Peroxidase Substrate (KPL Inc., Gaithersburg, MD, USA) and 1 N phosphoric acid. The plate was washed with 0.05% Tween-containing PBS between each treatment. The stabilities of the protein function and BG–BGRP binding were evaluated by treating the solid-phase supBGRP or 500 ng/mL LAM-conjugated solid-phase supBGRP with several aqueous solutions of different pH values or NaOH concentrations. The aqueous solutions used for the pH study were HCl (pH 0, 1), 0.05 M KCl-HCl buffer (pH 2), Macllvaine buffer (pH 3, 4, 5), phosphate buffer (pH 6, 7, 8, 11, 11.5, 12), Tris-HCl (pH 9), glycine-NaOH (pH 9.5, 10, 10.5), and NaOH (pH 13, 14). The pH of each solution was adjusted with Seven Easy (Mettler Toledo, Columbus, OH, USA).

### 4.4. Sample Preparation

JCP was kindly provided by Torii Pharmaceutical Co., Ltd. (Tokyo, Japan). In brief, 5 g of the pollen was stirred in 1 L of an aqueous 0.1 M NaHCO_3_ solution using a magnetic stirrer for 30 min at 25 °C. The suspension was then centrifuged at 6080× *g* and 10,080× *g* for 10 min. The collected supernatant was then filtered through a 0.20 μm PES bottle top filter (Thermo Fisher Scientific, Waltham, MA, USA). For preparation of the CA sup, *Candida albicans* (NBRC 1385) was first subcultured on a yeast extract-peptone-dextrose agar plate at 37 °C. A single colony was then picked and suspended in 5 mL of RPMI 1640 medium (Gibco, Gaithersburg, MD, USA) and incubated at 37 °C for 8 h. Thereafter, the yeast concentration was adjusted to 1 × 10^6^ cells/mL in RPMI 1640 medium, and 1 mL of the suspension was added to 20 mL of RPMI 1640 medium containing 10% fetal bovine serum (Biosera, Nueille, France). After 24 h incubation, the culture suspension was centrifuged at 3000× *g* for 10 min and the supernatant was filtered with a 0.45 μm syringe filter (Iwaki, Tokyo, Japan).

### 4.5. Beta-Glucan Recognition Protein Column Purification

To prepare the BGRP column, a Hitrap NHS-activated column (Cytiva, Marlborough, MA, USA) was conjugated with supBGRP according to the manufacturer’s protocol. Before its use for BG purification, the BGRP column was washed with 10 mL of PBS that was flowed through with a peristaltic pump set at 2 mL/min. Then, 900 mL of JCP extract or 100 mL of CA sup was passed through the column at 2 mL/min. After washing the column with 10 mL of PBS (wash fluid), the BG was eluted as five fractions (900 μL/fraction) using 0.03 M NaOH. The eluates were immediately neutralized with 300 μL of 0.1 M phosphate citrate buffer (pH 3.0). After elution, the column was washed with 10 mL of PBS. Finally, the liquid in the column was replaced with PBS containing Proclin 250 (Sigma-Aldrich) and the column system was stored at 4 °C.

## Data Availability

Data is contained within the article.

## Abbreviations

APBG	Beta-glucan from Aureobasidium pullulans
AP-FBG	Fermented beta-glucan from *Aureobasidium pullulans*
Bal	Barley beta-glucan
BG	Beta-1,3-d-glucan
BGRP	Beta-glucan recognition protein
CA	Candida albicans
CA sup	*Candida albicans* culture supernatant
CSBG	*Candida* solubilized beta-glucan
CMC	Carboxymethylcellulose
Curd	Curdlan
Dex	Dextran
DIW	Deionized water
EIA	Enzyme immunoassay
ext	Extract
JCP	Japanese cedar pollen
LAL	Limulus amebocyte lysate
LAM	Laminarin
LNT	Lentinan
Man	Mannan from Saccharomyces cerevisiae
mDectin-1	Mouse Dectin-1
Pach	Pachyman
Para	Paramylon
PBS	Phosphate-buffered saline
PEG	Polyethylene glycol
Pul	Pullulan
Pus	Pustulan
SCL	Scleroglucan
SPG	Schizophyllan
supBGRP	Artificial beta-glucan recognition protein
Xyl	Xylan from corn core
